# Efficacy of a lung recruitment manoeuvre in children undergoing general anaesthesia with a supraglottic airway

**DOI:** 10.1016/j.bja.2025.08.016

**Published:** 2025-09-23

**Authors:** Ricarda Lippuner, Charlotte Pellaud, Markus Huber, Robert Greif, Nicola Disma, Thomas Riva, Alexander Fuchs, Thomas Riedel

**Affiliations:** 1 Department of Anaesthesiology and Pain Medicine, Bern University Hospital, University of Bern, Bern, Switzerland; 2 Division of Paediatric Intensive Care Medicine, Department of Paediatrics, Inselspital, Bern University Hospital, University of Bern, Bern, Switzerland; 3 Medical Faculty, University of Bern, Bern, Switzerland; 4 Unit for Research in Anaesthesia, IRCCS Istituto Giannina Gaslini, Genova, Italy

**Keywords:** airway pressure, anaesthesia, atelectasis, lung volume, paediatrics, recruitment manoeuvres, respiratory physiology

## Abstract

**Background:**

Lung atelectasis is a well-recognised consequence of general anaesthesia in children and can cause poor oxygenation. Lung recruitment manoeuvre has been proposed as a strategy to counteract atelectasis. In this feasibility study, we evaluated whether a recruitment manoeuvre in children with a supraglottic airway restores end-expiratory lung volume (EELV) to baseline using electrical impedance tomography.

**Methods:**

After ethics committee approval (BASEC 2022-01833), we included children (ASA physical status 1–2, 10–20 kg) scheduled for anaesthesia with a supraglottic airway. We performed a recruitment manoeuvre by setting an anaesthesia machine to spontaneous breathing with a 3 L min^−1^ fresh gas flow (fraction of inspired oxygen [Fio_2_] 1.0) and the adjustable pressure-limiting (APL) valve set to 30 cmH_2_O, until a pressure plateau was reached or a leak occurred. Data were collected at three time points: after induction of anaesthesia (baseline), after placement of a supraglottic airway, and after recruitment manoeuvre. The primary endpoint was the normalised change in end-expiratory lung impedance after the recruitment manoeuvre. The global inhomogeneity index was a secondary outcome. Equivalence was assumed if the 95% confidence interval for estimated ΔEELV remained within ±35% (±2 ml kg^−1^).

**Results:**

Of the 95 eligible children, we analysed 74 datasets. The median (95% confidence interval) estimated difference in EELV after recruitment manoeuvre *vs* baseline was −0.9 ml kg^−1^ (−1.6 to −0.1), within the equivalence margin. No significant changes in global inhomogeneity index (0.0 [0.03–0.04]) were observed.

**Conclusions:**

In children undergoing general anaesthesia with the use of a supraglottic airway, a recruitment manoeuvre effectively restores EELV to baseline.

**Clinical trial registration:**

NCT05672329

**Editor’s key points**

• Children undergoing surgery under general anaesthesia frequently have postoperative lung atelectasis.
• Lung recruitment manoeuvres can be effective in reducing the incidence and the degree of postoperative lung atelectasis, but their effectiveness, particularly via a supraglottic airway, is not clear.
• This study shows that in anaesthetised children, a recruitment manoeuvre via a supraglottic airway is effective in reducing lung atelectasis, as the manoeuvre can restore end-expiratory lung volume to the baseline value.

Atelectasis, or the collapse of alveoli, is a well-recognised consequence of general anaesthesia in children, significantly impairing oxygenation and lung mechanics.^1^ Paediatric patients are particularly vulnerable because of their unique respiratory physiology, including higher chest wall compliance, lower functional residual capacity, and a greater tendency for airway collapse compared with adults.^2^ Atelectasis can lead to postoperative pulmonary complications such as hypoxaemia, impaired gas exchange, and pneumonia. Such complications may prolong hospital stay, highlighting the importance of preventing and treating atelectasis in paediatric anaesthesia. One proposed strategy to counteract atelectasis is the use of a lung recruitment manoeuvre, which involves a transient increase in airway pressure to reopen collapsed alveoli and improve ventilation.^3^ However, the effectiveness of recruitment manoeuvres in restoring end-expiratory lung volume (EELV), particularly when using a supraglottic airway, remains uncertain.

Supraglottic airways are widely used in paediatric anaesthesia because of their advantages over tracheal tubes, including reduced airway trauma and lower rates of laryngospasm.^4^^,^^5^ However, at higher airway pressures, potentially reducing the efficacy of recruitment manoeuvres or increasing the risk of gastric insufflation, gas may leak around a supraglottic airway.^6^ These concerns are especially relevant in children, owing to their smaller airway calibres and lower airway sealing pressures. Despite these limitations, some studies suggest that, in adults, modified recruitment strategies can be effective while a supraglottic airway is in place, although evidence remains limited.^7^

To evaluate recruitment manoeuvres, a reliable imaging technique is essential. Electrical impedance tomography (EIT) is a noninvasive, radiation-free method to measure changes in lung impedance (a proxy for changes in lung volume), regional lung ventilation, and tidal volume distribution.^8^

This study, which is a secondary analysis of a previously published randomised controlled trial,^9^ represented a feasibility study propaedeutic to a future randomised controlled trial and aimed to evaluate whether a recruitment manoeuvre in children under general anaesthesia and a supraglottic airway restores baseline breathing conditions. We hypothesised that a recruitment manoeuvre after placement of a supraglottic airway would restore EELV to the baseline lung volume after induction of anaesthesia.

## Methods

After approval from the responsible Cantonal Ethics Committee of Bern (BASEC 2022-01833) and registration at ClinicalTrials.gov (NCT05672329), we obtained written informed consent from the parents or legal guardians of all children before study enrolment. This study was conducted at the Department of Anaesthesiology and Pain Medicine, Bern University Hospital, Bern, Switzerland, from January 2023 to April 2024.

We included paediatric patients requiring general anaesthesia for elective surgery in the operating room. The inclusion criteria were a body weight of 10–20 kg and ASA physical status of 1–2. The exclusion criteria were known or suspected difficult intubation, oxygen dependency, congenital heart or lung disease, BMI >30 kg m^−2^, and high risk of pulmonary aspiration. In this secondary analysis, we included all participants with supraglottic airways as the final airway strategy.

A detailed description of the study protocol has been published previously.^9^ In this analysis, children were premedicated with 0.5 mg kg^−1^ midazolam (oral or rectal) or 2 μg kg^−1^ dexmedetomidine (nasal) 30 min before entering the operating room. All patients were monitored according to the local standard of anaesthesia care, including peripheral oxygen saturation (Spo_2_), electrocardiogram, and non-invasive blood pressure. We applied TCM 5 (Radiometer, Krefeld, Germany) to measure transcutaneous carbon dioxide (PtCO_2_), near-infrared spectrometry (NIRS) with SensSmart X-100 (Nonin Medical B.V., Tilburg, the Netherlands), and a thoracic EIT belt (Pulmo Vista 500; Draeger, Luebeck, Germany). This belt comprises 16 electrodes plus one reference electrode and was fitted around the chest in a thoracic median plane. Intravenous access was established, and general anaesthesia was induced with fentanyl 2 μg kg^−1^ and propofol 2–3 mg kg^−1^. In case intravenous access was difficult to obtain, anaesthesia was induced with inhaled sevoflurane followed by the placement of an intravenous cannula. General anaesthesia was maintained using propofol 10–15 mg kg^−1^ h^−1^. Depth of anaesthesia was assessed with Narcotrend™ (Narcotrend, Hannover, Germany), maintaining values < 60. All participants received rocuronium 0.9 mg kg^−1^. A train-of-four (TOF) of 0 monitored with a TOF-Watch (Organon Ltd, Dublin, Ireland) ensured complete neuromuscular block during study measurements.

One minute of pressure-controlled mask ventilation (Fio_2_ 1.0, Pmax 20 cmH_2_O, backup frequency 20 min^−1^, positive end-expiratory pressure [PEEP] 5 mm Hg) was applied, aiming for a tidal volume of 6 ml kg^−1^. After the TOF was 0, the ventilation was discontinued, and the child was left apnoeic for 5 min with Fio_2_ 1.0 in a supine position. The apnoeic period ended after 5 min or earlier if any of the following termination criteria were met: (1) decrease of Spo_2_ <95%; (2) increase of PtCO_2_ >70 mm Hg; or (3) decrease of NIRS of more than 30% from baseline. During the apnoeic period, each child received apnoeic oxygenation with a high-flow oxygen device (Optiflow, Fisher & Pykel Healthcare, Auckland, New Zealand).

After the apnoeic period, a second-generation supraglottic airway (Ambu AuraGain size 2, Ambu A/S, Ballerup, Denmark) was placed, and the recruitment manoeuvre carried out. The ventilator (Primus, Draeger, Luebeck, Germany) was set in spontaneous breathing mode with a fresh gas flow of 3.0 L min^−1^ (Fio_2_ 1.0), and the adjusting pressure-limiting (APL) valve was set to 30 cm H_2_O. The manoeuvre continued until a pressure plateau was reached or a leak occurred. After the recruitment manoeuvre, pressure-controlled ventilation was applied with a tidal volume of 6 ml kg^−1^.

A chest ultrasound after the end of induction ruled out pneumothorax (Venue Go; GE Healthcare, Chicago, IL, USA).

We recorded thoracic EIT measurements continuously at a frame rate of 30 Hz during the study period, starting when the child was still breathing spontaneously before induction of anaesthesia and ending 1 min after the recruitment manoeuvres. We excluded patient data in cases of EIT malfunction. We reconstructed EIT images from the raw data based on the Graz consensus reconstruction algorithm for EIT using the torso mesh function.^10^^,^^11^ The change in EIT from start of induction of anaesthesia to the end of recruitment manoeuvre was estimated by calculating lung impedance change, normalised to the impedance amplitude during mechanical ventilation at 6 ml kg^−1^ using adapted customised code (Matlab R2021a; The MathWorks Inc., Nattick, MA, USA).^12^^,^^13^ We divided each analysis of the EIT signals for each patient into three periods that represent time: (1) spontaneous breathing (patient pre-oxygenated and spontaneously breathing), (2) baseline (patient’s lungs ventilated with a mask after induction), and (3) after (immediately after recruitment manoeuvre). Each period included a minimum of five breaths.

### Statistical analysis

The primary outcome was the total estimated change in EELV normalised to the impedance amplitude during mechanical ventilation at 6 ml kg^−1^ after recruitment manoeuvre compared with a baseline during induction of anaesthesia immediately before apnoea measured by EIT. The secondary outcomes included the changes in global inhomogeneity (GI) index before induction, during spontaneous breathing, and after recruitment manoeuvre and comparing the estimated change in EELV from spontaneous breathing with after recruitment manoeuvre. Categorical data were summarised with counts and frequencies.

Numerical data were summarised with the mean and standard deviation in cases of normally distributed variables and with the median and interquartile range otherwise. The primary outcome was tested using an equivalence framework with a predefined equivalence margin: If the two-sided 95% confidence interval (CI) (corresponding to a significance level of *α*=0.025) for the difference in global reduction in lung impedance lay entirely within the predefined equivalence margins of within 35%, equivalence was assumed. This corresponds to a difference in estimated lung volume of 2 ml kg^−1^ being considered equivalent. We considered the median and the associated 95% CI of the median to assess equivalence. The 95% CI for a paired-samples median difference was calculated with the statpsych package.^14^

## Results

**Fig 1:**
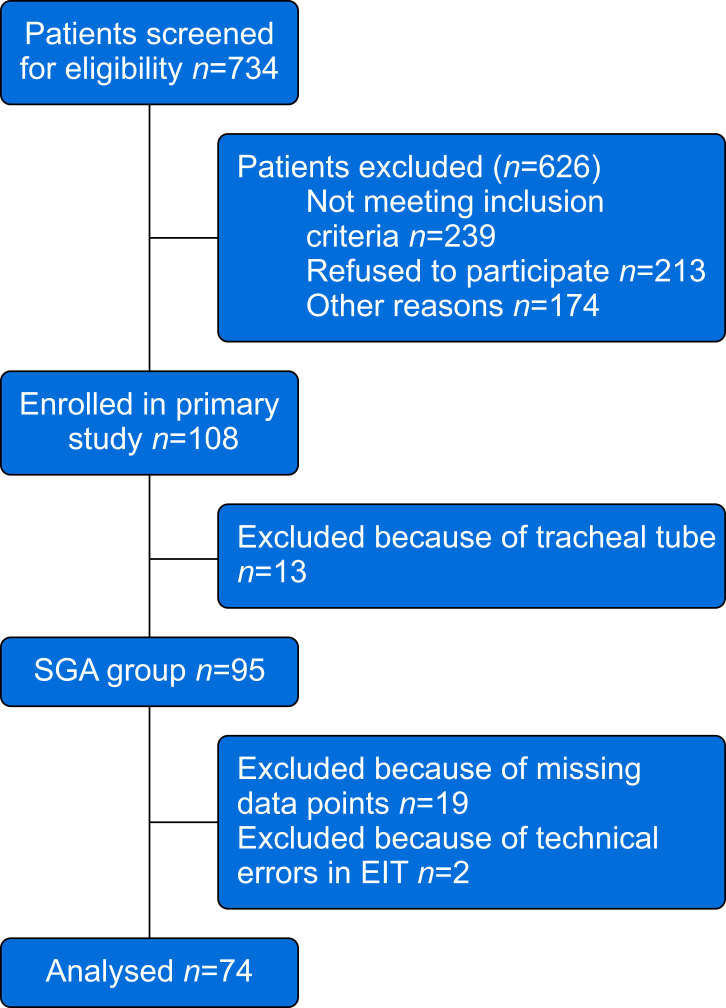
Study flowchart. Flow diagram illustrating the number of screened patients, reasons for exclusion, number of enrolled, and analysed participants. EIT, electrical impedance tomography; SGA, supraglottic airway.

For this secondary analysis, 95 participants were eligible, and 74 patients went into the final analysis. We excluded 21 patients: 19 for missing data and two for EIT technical errors (Fig. 1).

**Fig 2:**
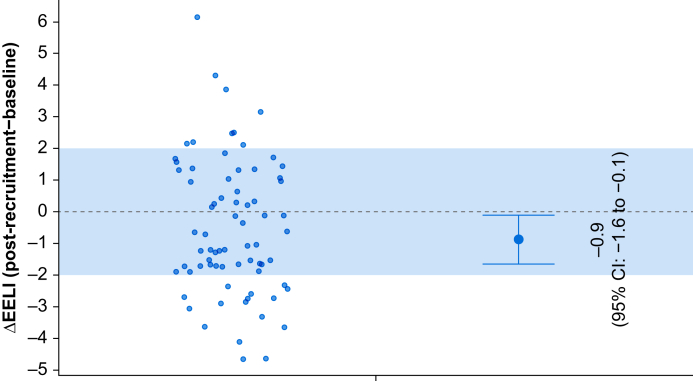
Individual and median changes in end-expiratory lung impedance (EELI) after the recruitment manoeuvre compared with baseline. Each dot represents an individual patient’s ΔEELI (post-recruitment minus baseline). The shaded area denotes the predefined margin of clinical equivalence (±35%). The black dot with error bars on the right shows the median ΔEELI with 95% confidence interval (CI). The entire CI lies within the equivalence margin, suggesting clinical equivalence.

The estimated mean (95% CI) difference in EELV after the recruitment manoeuvre compared with the baseline value was −0.9 ml kg^−1^ (−1.6 to −0.1) (*P*=0.03). As the difference was smaller than 2 ml kg^−1^, recruitment manoeuvres with a supraglottic airway effectively restored lung volume to baseline values (Fig. 2).

**Fig 3:**
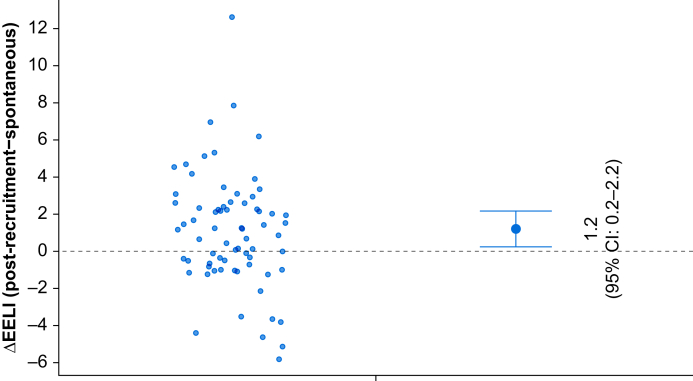
Individual changes in end-expiratory lung impedance (EELI) after the recruitment manoeuvre compared with the spontaneous breathing reference phase. Each dot represents an individual patient’s ΔEELI (post-recruitment minus spontaneous). Positive values indicate an increase in lung volume after the recruitment manoeuvre. The dashed line at 0 represents no change from the spontaneous breathing baseline. 95% CI, 95% confidence interval.

When comparing EELV after the recruitment manoeuvre with spontaneous breathing, the estimated mean difference was 1.2 ml kg^−1^ (0.2–2.2) (*P*=0.003) (Fig. 3).

No significant differences in the GI index were observed when comparing after the recruitment manoeuvre with the baseline (0.0 [0.00–0.01]), indicating preserved ventilation distribution.

More homogeneous ventilation was observed after the recruitment manoeuvre compared with spontaneous breathing (−0.05 [−0.09 to −0.01], *P*<0.001) (Table 1).

**Table 1:** Summary of end-expiratory lung impedance (EELI) and global inhomogeneity (GI) index across ventilation phases. Data are presented as median (95% confidence interval). EELI values are expressed as changes relative to the post-recruitment reference phase (ΔEELI), which was set to 0 by definition. Compared with post-recruitment, EELI values were lower during spontaneous breathing (−1.19 [−2.53 to 0.70]) and higher at baseline (0.87 [−1.07 to 1.84]).

	*n*=74
EELIspont	−1.19 (−2.53 to 0.70)
EELIbaseline	0.87 (−1.07 to 1.84)
EELI post-recruitment (by definition)	0
GI spont	0.59 (0.56–0.62)
GI baseline	0.55 (0.53–0.58)
GI post-recruitment	0.56 (0.53–0.59)

## Discussion

This feasibility study and secondary analysis of data from a prospective single-centre study have shown that recruitment manoeuvre after placement of a supraglottic airway in anaesthetised children can restore the estimated EELV to the baseline set before apnoea. Our findings suggest that a recruitment manoeuvre via a supraglottic airway may help reopen collapsed alveoli, restore the EELV slightly below baseline conditions, and enhance lung mechanics in children. Clinicians may consider incorporating the recruitment manoeuvre into their clinical routine after placement of a supraglottic airway.

The restoration of EELV was primarily attributable to the transient increase in airway pressure generated during the recruitment manoeuvre. Although apnoeic oxygenation was applied at varying flow rates, the FUTURE trial^9^ demonstrated a rapid loss of lung volume within seconds after the onset of apnoea, regardless of the nasal oxygen flow rate. Before the application of the recruitment manoeuvre, the estimated loss of lung volume was around 6 ml kg^−1^.^9^ These findings indicated that high-flow nasal oxygen did not produce sufficient positive airway pressure to prevent atelectasis formation. Therefore, the observed recovery in estimated EELV is a direct effect of the recruitment manoeuvre.

Given the high incidence of atelectasis in paediatric anaesthesia,^2^^,^^3^^,^^9^ various strategies have been developed to counteract its effects. Recruitment manoeuvres are among the most effective interventions and are typically applied in intubated patients. Tusman and colleagues^15^ demonstrated that pressures must exceed the critical opening pressure of collapsed alveoli to achieve effective recruitment, with levels up to 40 cmH_2_O shown to be well tolerated in children.^16^^,^^17^ These findings challenge the long-held assumption that supraglottic airways are unsuitable for lung recruitment and highlight their potential as a practical and effective tool in paediatric anaesthesia.^18^ Recruitment manoeuvres via supraglottic airways can deliver sufficient airway pressure to reopen collapsed alveoli, even with minor leaks. This provides a simple method to improve perioperative lung mechanics. This is especially relevant because supraglottic airways are the most common airway device used in paediatric patients.^5^

Key factors likely contributing to a successful recruitment manoeuvre with supraglottic airways included the use of a second-generation supraglottic airway with good sealing (Ambu AG, Lerzenstrasse 108953 Dietikon), a standardised approach with a 3 L min^−1^ fresh gas flow and an APL valve at 30 cmH_2_O, and timing the manoeuvre immediately after apnoea and airway management, when lung volume loss was likely greatest. Additionally, our population of otherwise healthy children with ASA 1–2 physical status scores, who received full neuromuscular block, may have contributed to the responsiveness of the lungs.

Analysis of the GI index revealed no significant difference after the recruitment manoeuvre compared with baseline. This demonstrates that ventilation distribution was preserved after the recruitment manoeuvre.

When comparing the GI index after the recruitment manoeuvre with spontaneous breathing, a significant improvement was observed. This may be attributable to premedication (benzodiazepines or dexmedetomidine), which might already impact breathing patterns before induction.

Finally, the use of EIT allowed sensitive and objective assessment of lung volume changes, potentially capturing recruitment effects more accurately than traditional methods. This simple and reproducible technique can be applied in similar paediatric settings to achieve comparable results. Nevertheless, clinicians should remain mindful of patient-specific factors, device variability, and the need to monitor for complications such as hypotension, desaturation, or barotrauma.^19^ EIT can be used to monitor perioperative lung volume loss.^20^^,^^21^ However, there is still limited research on using EIT to assess recruitment manoeuvres in children. Most available data come from animal studies.^22^ The findings of this study underscore the potential for EIT-guided strategies to optimise perioperative lung function, particularly in healthy children undergoing routine procedures. Further studies are warranted to explore the bedside use of EIT for assessing recruitment manoeuvres, aiming to improve oxygenation while maintaining stable haemodynamic. This has been explored in adult settings^23^ and should be extended to healthy children and eventually even to children at risk for perioperative complications.

Limitations of this study are the single-centre study design and the lack of randomisation, which limits generalisability. The lack of randomisation and the comparison with a standardised recruiting manoeuvre through a tracheal tube represent significant limitations.

Although EIT offers a radiation-free method for evaluating lung impedance changes, it is prone to artifacts, especially when used during apnoea, as it was initially designed to measure tidal volume. The apnoea phase was not part of the presented analysis, so this should not be relevant for our results. No direct measurements of tracheal pressure were taken because of safety concerns in paediatric patients. Furthermore, participants were otherwise healthy children with no risk factors for postoperative pulmonary complications. Participants with ASA 3–4 were excluded, limiting applicability to higher-risk patients and potentially introducing selection bias. However, the vast majority of children undergoing anaesthesia for surgical procedures are ASA 1–2 and otherwise healthy, and supraglottic airways are largely used for ventilation in these patients. Our findings are then easily applicable in daily practice with no risk of harm to patients. In the most vulnerable population, those with respiratory comorbidities, recruiting manoeuvres may represent a rescue technique for restoring adequate oxygenation; however, further research is still needed. Another limitation is that 21 children had to be excluded from the analysis because of missing measurements or technical errors with the EIT. However, the patients characteristics of the excluded patients did not differ significantly from those of the patients included in the analysis. Given the relatively narrow CI of our primary outcome, we do not expect the inclusion of these patients would have altered the results.

In conclusion, in children undergoing general anaesthesia with supraglottic airway, a recruitment manoeuvre performed with the anaesthesia machine effectively reopens collapsed alveoli and restores EELV to baseline without increasing ventilation inhomogeneity. These findings challenge the assumption that supraglottic airways are unsuitable for lung recruitment, highlight their practical value in paediatric anaesthesia, and should be further explored, especially in vulnerable or high-risk paediatric patients.

## Authors’ contributions

Project administration, funding acquisition, validation, investigation, resources, formal analysis: TRie, TRiv, AF

Conceptualisation, methodology, writing, review and editing of the manuscript: TRie, TRiv, AF, RG, ND

Data curation, writing of the original draft: RL, CP

Formal analysis: MH

## Funding

Department of Anaesthesiology and Pain Medicine, University Hospital of Bern, Bern, Switzerland; Burgergemeinde Bern (grant to TRiv).

## Declaration of interest

The authors declare that they have no conflicts of interest.
